# Building Community and Belonging: Launch of the First Face-to-Face International Medical Graduate (IMG) Networking Conference in the West Midlands

**DOI:** 10.1192/bjo.2026.11448

**Published:** 2026-06-30

**Authors:** Omotola Ogunjobi, Asma Javed, Mohammed Elgohary

**Affiliations:** 1 Black Country Healthcare NHS Foundation Trust, Dudley, United Kingdom; 2 Birmingham and Solihull Mental Health Foundation Trust, Birmingham, United Kingdom

## Abstract

**Aims:**

This inaugural face-to-face IMG conference aimed to foster belonging, strengthen peer connections, and provide a supportive networking environment for IMGs across the West Midlands. It also sought to understand attendees’ expectations and gather structured feedback on their experience.

IMG trainees constitute approximately 50% of the psychiatry training cohort in the West Midlands. GMC progression reports indicate that IMGs experience differential attainment in examinations and training. In the West Midlands, we are committed to reducing this attainment gap by offering support and guidance from the outset. TPDs and Tutors aim to meet trainees at induction and subsequently every 3–4 months via Microsoft Teams along with 1:1 meeting.

This year, we have developed an initiative to launch the first West Midlands IMG Conference, designed to strengthen community and foster a shared identity among IMGs. A regional in-person event was identified as an effective way to bring trainees together, provide access to experienced speakers, and create a relaxed environment for meaningful interaction to enhance belonging and support.

**Methods:**

Email invitations were sent to all IMGs across the region with support from the Future Workforce Team at the School. Registration was managed using an online Microsoft Form link. We secured a room within the trust at no additional cost, allowing the conference to remain free for IMG attendees. All 30 available places were filled within days, with further expressions of interest received. Registrants were sent personalised reminders 14, 7, and 2 days before the event.

The TPD and the IMG tutors coordinated logistics, speaker engagement, refreshments, and the overall attendee experience. The programme included sessions on purposeful leadership, psychotherapy, Less-Than-Full-Time Training, and an account of being an IMG trainee in the West Midlands. Icebreaker activities, including brief introductions and a charades game, were used to encourage early engagement.

**Results:**

Twenty core and higher psychiatry trainees attended. The atmosphere was energetic with networking developing naturally after the icebreakers.

Eleven attendees completed feedback forms, giving a mean satisfaction score of 4.73/5. Comments emphasised the positive atmosphere, supportive intent, enjoyable activities, and valuable opportunities for networking.

**Conclusion:**

IMGs value dedicated spaces that promote connection, learning, and wellbeing. This event enabled participants to feel recognised and empowered, reinforcing the need for similar future initiatives.

